# Left‐handed musicians show a higher probability of atypical cerebral dominance for language

**DOI:** 10.1002/hbm.24929

**Published:** 2020-02-07

**Authors:** Esteban Villar‐Rodríguez, María‐Ángeles Palomar‐García, Mireia Hernández, Jesús Adrián‐Ventura, Gustau Olcina‐Sempere, María‐Antònia Parcet, César Ávila

**Affiliations:** ^1^ Neuropsychology and Functional Neuroimaging Group Jaume I University, Edificio de Investigación II Castellón de la Plana Spain; ^2^ Cognition and Brain Plasticity Group, Department of Cognition, Development and Educational Psychology, Institut de Neurociències University of Barcelona Barcelona Spain

**Keywords:** functional laterality, hemispheric language dominance, lateralization, left‐handedness, musicians

## Abstract

Music processing and right hemispheric language lateralization share a common network in the right auditory cortex and its frontal connections. Given that the development of hemispheric language dominance takes place over several years, this study tested whether musicianship could increase the probability of observing right language dominance in left‐handers. Using a classic fMRI language paradigm, results showed that atypical lateralization was more predominant in musicians (40%) than in nonmusicians (5%). Comparison of left‐handers with typical left and atypical right lateralization revealed that: (a) atypical cases presented a thicker right pars triangularis and more gyrified left Heschl's gyrus; and (b) the right pars triangularis of atypical cases showed a stronger intra‐hemispheric functional connectivity with the right angular gyrus, but a weaker interhemispheric functional connectivity with part of the left Broca's area. Thus, musicianship is the first known factor related to a higher prevalence of atypical language dominance in healthy left‐handed individuals. We suggest that differences in the frontal and temporal cortex might act as shared predisposing factors to both musicianship and atypical language lateralization.

## 1 INTRODUCTION

Since the postmortem findings from the studies by Broca (Broca, 1861) and Wernicke (Wernicke, 1874), we know that the language function is mainly left‐lateralized (mostly processed by the left hemisphere). Modern techniques such as Wada testing and fMRI assessment have allowed us to confirm this, while also making some clarifications: (a) it is only applicable to ~94–96% of the right‐handed population (Pujol, Deus, Losilla, & Capdevila, 1999; Springer et al., 1999) because the left‐handed population presents a lower incidence of typical left dominance, ~76–78% (Mazoyer et al., 2014; Pujol et al., 1999; Szaflarski et al., 2002); (b) in the left‐handed population, atypical dominance consists of both bilateral cases (~14–15%) and right‐lateralized cases (~7–10%; Mazoyer et al., 2014; Pujol et al., 1999; Szaflarski et al., 2002); and (c) some clinical conditions such as temporal lobe epilepsy, schizophrenia, and early left‐hemisphere lesions have been related to higher incidences of atypical (right or bilateral) language lateralization (Müller et al., 1999; Sommer, Ramsey, & Kahn, 2001; Springer et al., 1999; Stewart et al., 2014). Therefore, we know that hemispheric dominance of language is subject to modulation by different genetic and early lesional factors.

The brain bases underlying language lateralization and its modulation in the healthy population have historically been thought to be dependent on structural differences present in the temporal lobe. The logic behind this idea is the marked leftward hemispheric asymmetry present in some temporal regions and the importance of these regions for speech processing (Geschwind & Levitsky, 1968). However, current neuroimaging morphometry procedures have been inconsistent when trying to prove this relationship, revealing both negative (Dorsaint‐Pierre et al., 2006; Greve et al., 2013; Keller et al., 2011; Tzourio‐Mazoyer, Crivello, & Mazoyer, 2018) and positive reports (Greve et al., 2013; Keller et al., 2018; Sreedharan, Menon, James, Kesavadas, & Thomas, 2015; Tzourio‐Mazoyer et al., 2015). Positive findings on the relationship between the temporal lobe structure and functional lateralization of language in healthy populations have been observed in: (a) gray matter surface hemispheric asymmetry of the planum temporale or secondary auditory cortex (Greve et al., 2013); (b) hemispheric asymmetry of arcuate fasciculus volume (Sreedharan et al., 2015); and (c) the gyri duplication pattern of Heschl's gyrus (HG) or primary auditory cortex (Tzourio‐Mazoyer et al., 2015). The pars triangularis of the inferior frontal gyrus (IFG), or Broca's area, has also been a focus of lateralization research, with some studies pointing to its rightward asymmetry as a marker of rightward language lateralization (Foundas, Leonard, Gilmore, Fennell, & Heilman, 1996; Josse, Kherif, Flandin, Seghier, & Price, 2009; Keller et al., 2018).

The way these structural asymmetries might influence the development of language dominance also continues to be a relevant question. Perisylvian asymmetries have been found prenatally in both neuroimaging (Kasprian et al., 2011) and postmortem studies (Wada, Clarke, & Hamm, 1975). In line with these early structural biases, speech processing has been found to be leftward asymmetrical as early as the age of 3 months in the superior temporal gyrus and angular gyrus (Dehaene‐Lambertz, Dehaene, & Hertz‐Pannier, 2002), and at the age of 6 months in the IFG (Imada et al., 2006). However, longitudinal assessment of language dominance revealed that, at least from 5 to 11 years old, language lateralization increases as a function of age, especially in Broca's area and Wernicke's area (Szaflarski et al., 2006). In fact, functional connectivity patterns between language areas have been described as fundamentally different in adults (more intra‐hemispheric) when compared to children (more interhemispheric; Friederici, Brauer, & Lohmann, 2011). Thus, even if we assume a possible causal role of structural asymmetries in language dominance development, there still seems to be room for other not‐so‐ early factors to influence and significantly modulate lateralization.

Considering the aforementioned mechanism for the development of language lateralization, we wondered if musicianship could be related to a higher incidence of atypical language dominance in left‐handers. Our proposal is rooted in the fact that musicianship has been associated with differences in the right auditory and frontal cortices (as well as their connections), brain regions whose possible involvement in language lateralization has also been described. Some of the most overlapping findings between musicians and right‐lateralized persons are: (a) greater gray matter density and volume in the right auditory cortex in musicians (Bermudez, Lerch, Evans, & Zatorre, 2009; Palomar‐García, Zatorre, Ventura‐Campos, Bueichekú, & Ávila, 2017) and rightward asymmetry of a portion of the auditory cortex in right‐lateralized persons (Greve et al., 2013); (b) a higher incidence of duplications in both HG in musicians (Benner et al., 2017) and a loss of leftward language lateralization when the left HG is duplicated (Tzourio‐Mazoyer et al., 2015); (c) a higher fractional anisotropy in the right arcuate fasciculus in musicians (Halwani, Loui, Rüber, & Schlaug, 2011) and a rightward asymmetry of arcuate fasciculus volume in right‐lateralized persons (Sreedharan et al., 2015); and (d) a thicker cortex in the right pars triangularis in musicians, correlating positively with pitch discrimination ability in nonmusicians (Bermudez et al., 2009; Novén, Schremm, Nilsson, Horne, & Roll, 2018), and a rightward asymmetry of Broca's area in right‐lateralized persons (Foundas et al., 1996; Josse et al., 2009; Keller et al., 2018). In fact, language and music are processes whose relationship has been demonstrated through interactions at the subcortical level of processing (Musacchia, Sams, Skoe, & Kraus, 2007) and the performance of some language skills (Slater & Kraus, 2016). Thus, given the similarity in the neuroanatomical correlates of the two conditions, we hypothesized that atypical language dominance would be found more frequently in left‐handed musicians than among left‐handed nonmusicians. Because anatomical differences associated with musicianship emanate not only from musical training, but are also thought to depend on preexisting factors (Foster & Zatorre, 2010; Vaquero, Ramos‐Escobar, François, Penhune, & Rodríguez‐Fornells, 2018), we think the predicted association would be related to both predisposing and experiential factors.

Hence, the objective of this study was to test whether left‐handed musicians presented differences in hemispheric language lateralization compared to left‐handed nonmusicians. Because of overlapping neuroanatomical correlates between musicians and atypically lateralized persons in auditory and frontal cortices, we hypothesized that atypical lateralizations of language (right and bilateral) would be more prevalent among musicians. Differences between the left‐lateralized and right‐lateralized groups relevant to musical training (age of onset and approximate hours of training) were also explored in order to determine the relative contribution of experiential factors to this possible effect. As a second part of our study, we aimed to find brain correlates of language lateralization that would support this relationship between musicianship and hemispheric dominance. Based on the aforementioned overlapping anatomical correlates, we tested the following structural hypotheses: (a) greater cortical thickness in the right pars triangularis of right‐lateralized participants; (b) a higher gyrification index in the left HG of right‐lateralized participants; and (c) a greater gray matter volume in the right HG of right‐lateralized participants. Due to its possible relationship with language lateralization (Foundas et al., 1996; Greve et al., 2013; Josse et al., 2009; Keller et al., 2018), the hemispheric asymmetry of these neuroanatomical measures was also explored. Functionally, we explored lateralization‐related differences in: (a) brain activity during an expressive language task (task‐based fMRI); and (b) functional connectivity at rest in critical IFG areas for language (rs‐fMRI).

## 2 METHODS

### 2.1 Participants

Forty‐nine participants were included in the present study. All participants were left‐handed or ambidextrous, according to the Edinburgh Handedness Inventory/EHI (Bryden, 1977; Oldfield, 1971). We recruited non‐right‐handed participants in order to increase the likelihood of finding atypical lateralizations of language. Participants were classified into two groups according to their musical training: musicians (*n* = 30; 22 males; age mean ± *SD* = 20.4 ± 2.1; EHI score mean ± *SD* = 42.6 ± 5.1) and nonmusicians (*n* = 19; 11 males; age mean ± *SD* = 21.1 ± 2.3; EHI score mean ± *SD* = 40.6 ± 6.5). The conditions for being classified as a musician were having received musical training (at an official music school) for at least 9 years and currently playing a musical instrument (musical training duration mean ± *SD* = 13.1 ± 3.3 years). Nonmusicians, on the other hand, had never played a musical instrument or received musical training beyond basic school education. There were no statistically significant between‐group differences in gender (Fisher's exact test; *p* = .35), age (Mann–Whitney *U* = 234.5; *p* = .29), or EHI score (*t*_*47*_ = −1.21; *p* = .23). None of the participants had suffered any neurological or psychiatric disorders or had a history of head injury with loss of consciousness. Written informed consent was obtained from all participants following a protocol approved by the Jaume I University. All methods were carried out in accordance with approved guidelines and regulations.

### 2.2 fMRI paradigms: Verb generation task and resting‐state

For the evaluation of the expressive language function, we used a computerized verb generation task suited for MRI scanners (Sanjuán et al., 2010). In summary, it consists of a block design paradigm with two conditions: control and activation. During the control condition, letter pairs are visually and consecutively presented to the participant, who has to read them aloud. During the activation condition, concrete nouns are visually and consecutively presented to the participant, who has to say aloud the first verb that comes to mind for each noun. The task is composed of six control blocks and six activation blocks that are presented alternately. Stimuli were presented visually using MRI‐compatible goggles, and responses were recorded via a noise‐canceling microphone to verify that each participant was engaged correctly in the task. Before entering the scanner, participants practiced with a different version of the task for 2 min.

In order to study functional connectivity, participants underwent a resting‐state fMRI session (rs‐fMRI). During this paradigm, participants are instructed to just lie in the scanner with their eyes open and try not to sleep or think about anything in particular.

### 2.3 Image acquisition

Images were acquired on a 3‐T Philips Achieva scanner. A 3D structural MRI was acquired for each subject using a T1‐weighted magnetization‐prepared rapid gradient‐echo sequence (TR/TE = 8.4/3.8 ms; matrix = 320 × 320 × 250; voxel size = 0.75 × 0.75 × 0.8 mm). For the fMRI, 150 volumes (task) and 150 volumes (resting‐state) were recorded using a gradient‐echo T2*‐weighted echo‐planar imaging sequence (TR/TE of task = 2500/30 ms; TR/TE of resting‐state = 2000/30 ms; matrix = 80 × 80; voxel size = 3 × 3 × 4 mm). Thirty‐one interleaved axial slices were acquired, aligned to the plane that intersected the anterior and posterior commissures (AC–PC) and covered the whole brain.

### 2.4 Language lateralization analysis: Laterality index

Each participant was categorized according to his/her hemispheric pattern of language processing as: left‐lateralized, right‐lateralized, or bilateral. In order to classify them, we calculated their Laterality Index (LI) for the verb generation task. The first step in this procedure involved preparing the task‐fMRI data using the Statistical Parametric Mapping software package (SPM12; Wellcome Trust Centre for Neuroimaging, London, UK). Preprocessing followed the default pipeline and included: (a) alignment of each participant's fMRI data to the AC‐PC plane by using his/her own anatomical image; (b) head motion correction, where the functional images were realigned and resliced to fit the mean functional image; (c) co‐registration of the anatomical image to the mean functional image; (d) re‐segmentation of the transformed anatomical image; and (e) spatial normalization of the functional images to the MNI (Montreal Neurological Institute, Montreal, Canada) space with 3 mm^3^ resolution. In concordance with a previously described procedure (Sanjuán et al., 2010), functional images were not spatially smoothed because the following analysis involved counting the significantly active voxels. The general linear model of the verb generation task was defined for each participant by contrasting *activation condition > control condition*. The BOLD (Blood‐Oxygen‐Level‐Dependent) signal was estimated by convolving the task's block onsets with the canonical hemodynamic response function. Six motion realignment parameters extracted from head motion preprocessing were included as covariates of no interest. Lastly, a high‐pass filter (128 s) was applied to the functional data to eliminate low‐frequency components.

Next, contrast images were visualized for every subject using the iTT toolbox (iterative‐two‐threshold; Auer & Frahm, 2009) for SPM12. This two‐threshold approach first applies a strict upper threshold (*p* < .001) to all voxels, and it subsequently applies a less strict lower threshold (*p* < .05) to the voxels directly adjacent to the ones selected by the first threshold. As a result, the significant voxels obtained are defined by two different thresholds, thus lowering the impact of arbitrary selection of a statistical threshold in the calculation of laterality indexes (Seghier, 2008). As a final step, we applied an inclusive mask to the results in order to include only the significant voxels that are most relevant in the expressive language function evaluated by the task. We defined this mask using standard anatomical criteria (Rutten, Ramsey, van Rijen, & van Veelen, 2002), thus encompassing Brodmann areas 9, 44, 45, and 46. We used the Tailarach Daemon atlas (Lancaster et al., 2000; Lancaster, Summerlin, Rainey, Freitas, & Fox, 1997) included in the WFU PickAtlas toolbox (Maldjian, Laurienti, Kraft, & Burdette, 2003) to create the mask, inserting the areas with a 3D dilatation value of 2. Additionally, medial areas were subtracted by a boxcar with dimensions of 20, 100, 100, and an epicenter at 0, 0, 0. The resulting mask is shown in Figure S1.

Finally, the resulting image for each participant was used to calculate the LI through the formula: [(*L* – *R*)/(*L* + *R*)] × 100, where *L* and *R* are the number of significantly active voxels for the left and right hemispheres, respectively. Hence, LI is a proportion of the spatial extent of brain activity between the two hemispheres during this particular task, thus giving us information about the hemispheric lateralization of the expressive language function. The resulting LI values range from +100 (totally left‐lateralized function) to −100 (totally right‐lateralized function). Individual LI values were used to categorize participants according to criteria found in previous studies (Springer et al., 1999; Szaflarski et al., 2002): LI > +20 were categorized as left‐lateralized, LI < −20 were categorized as right‐lateralized, and LI ranging between −20 and + 20 were categorized as bilateral. In order to ensure that our lateralization classification was not biased by the use of the spatial extent of the activation rather than the magnitude of the activation (see Bradshaw, Bishop, & Woodhead, 2017), we double‐checked this assessment using an alternative approach to calculate LI that focuses on the magnitude of activation. We used the LI‐toolbox (Wilke & Lidzba, 2007) to apply an adaptive threshold procedure and calculate LI by using voxel values rather than voxel count. LIs calculated with both approaches were highly correlated (*r* = 0.742; *p* < .001), and they classified all the participants in the same manner. Hence, our lateralization classification was robust, regardless of the procedure selected for LI calculation. Homogeneous distribution of atypical patterns of language lateralization (right‐lateralized and bilateral) in musicians and nonmusicians was tested using Fisher's exact test. It should be noted that, in order to make the lateralization groups as homogenous as possible, the only participant categorized as bilateral was excluded from further analyses. Therefore, the following analyses were carried out with a sample consisting of 36 left‐lateralized participants (18 musicians) and 12 right‐lateralized participants (11 musicians). The only exception was the seed‐based resting‐state functional connectivity (rs‐FC) analysis, where we had to discard four left‐lateralized participants (one musician) because of excessive head motion during the data acquisition.

We also compared relevant variables related to musical training between left‐lateralized and right‐lateralized musicians. Variables examined were age of onset of musical training (years old; comparison via a Mann–Whitney's *U*) and amount of musical training (total hours; comparison via a two‐sample *t*_27_ test). Age of onset was self‐reported by each musician, whereas we calculated the amount of musical training based on self‐reports of how many hours per week they had practiced music during different age ranges. In order to compare the amount of musical training at equal ages across all musicians, we only contemplated the age range of 9–18 years old because the latest age of onset was 9 years, and the youngest musician was 18 years old.

### 2.5 Task‐fMRI analyses

The first dataset in which we explored lateralization‐related differences was the verb generation task. To this end, spatial smoothing (FWHM = 4 mm) was applied to the preprocessed task‐fMRI data previously used for the LI calculation. Afterwards, in order to know the brain regions involved during the task in the two differently lateralized groups, we used SPM12 to perform whole‐brain one‐sample *t* tests (voxel‐wise *p* < .001; FWE cluster‐corrected at *p* < .05) for left‐lateralized participants (*df* = 34) and right‐lateralized participants (*df* = 10), including musician/nonmusician as a dummy covariate of no interest. Then, we analyzed between‐group differences during the task, performing a whole‐brain two‐sample *t*_*45*_ test (voxel‐wise *p* < .001; FWE cluster‐corrected at *p* < .05), and including musician/nonmusician as a dummy covariate of no interest.

### 2.6 Structural analyses

Next, we tested our hypotheses about the structural correlates of language lateralization by applying both voxel‐based (VBM) and surface‐based (SBM) Region of Interest (ROI) analyses, using the CAT12 toolbox (http://www.neuro.uni-jena.de/cat/) for SPM12. VBM preprocessing included: (a) segmentation into gray matter, white matter, and cerebrospinal fluid; (b) registration to the ICBM standard template; (c) DARTEL normalization of gray matter segments to the MNI template; and (d) extraction of ROI‐values from native space. Lastly, we compared the gray matter volume of a ROI delimited by the voxels mapped as right HG, using the atlas provided by Neuromorphometrics, Inc. (http://neuromorphometrics.com/), which is freely distributed within the CAT12 toolbox. For the comparison, we used a one‐tailed two‐sample *t*_*43*_ test to find out whether right‐lateralized participants presented a higher volume in this region. Age, total intracranial volume, and musician/nonmusician were included as covariates of no interest. The standard pipeline for the SBM preprocessing described in the CAT12 manual was used, extracting both cortical thickness and the gyrification index. Afterwards, we analyzed the cortical thickness of the right pars triangularis and the gyrification index of the left HG using an ROI approach. Right pars triangularis ROI was defined based on the peak voxel from the right pars triangularis activation cluster from the verb generation task of right‐lateralized participants (see Figure S3). Hence, using a projection of the HCP‐MMP1 surface atlas (Glasser et al., 2016) to the MNI space (https://bit.ly/2aLrm9y), we selected the region of the atlas encompassing this voxel: the right IFSa (area 82). For the analysis of the left HG, we used the following regions classified by the atlas as the early auditory cortex: A1 (area 24), LBelt (area 174), and MBelt (area 173). The PBelt (area 124) and RI (area 104) were discarded because they are mainly confined to the planum temporale and the retro‐insular region, respectively. Finally, we used one‐tailed two‐sample *t*_*44*_ tests to find out whether right‐lateralized participants presented greater cortical thickness in the right IFSa ROI and a greater gyrification index in the left A1, LBelt, and MBelt ROIs. Age and musician/nonmusician variables were included as covariates of no interest. Following the standard procedure in CAT12, all surface tests were corrected for multiple comparisons using an FDR threshold of *p* < .05.

Additionally, we calculated five asymmetry indexes (AI) for all five structural measures (gray matter volume of HG; cortical thickness of IFSa; and gyrification of A1, MBelt, and LBelt). AI were calculated through the formula: [(*L – R)/*(*L + R*)], where L and R are the volume/thickness/gyrification units for the left and right hemisphere ROIs, respectively. Similar to LI, AI ranges from +1 (totally leftward asymmetry) to −1 (totally rightward asymmetry). Then, we compared the five AI between the left‐lateralized and right‐lateralized groups using 5 two‐tailed two‐sample *t*_*45*_ tests, including musician/nonmusician as covariate of no interest. The asymmetry pattern of the structural measures that showed significant between‐group differences in AI was further analyzed by contrasting the volume/thickness/gyrification in one hemisphere versus the other, separately for the left‐lateralized group (two‐tailed paired *t*_*34*_ test, including musician/nonmusician as covariate of no interest) and the right‐lateralized group (two‐tailed paired *t*_*11*_ test, not including musician/nonmusician as covariate due to multicollinearity). All the asymmetry tests were corrected for multiple comparisons using an FDR threshold of *p* < .05.

### 2.7 Resting‐state fMRI analyses

Lastly, we explored possible differences between left‐lateralized and right‐lateralized participants in the functional connectivity of the pars triangularis of IFG. For this purpose, we performed a seed‐based rs‐FC analysis of the resting‐state fMRI data. Data were preprocessed using the Data Processing Assistant for the Resting‐State toolbox (DPARSFA; Chao‐Gan & Yu‐Feng, 2010), which is based on SPM and the Data Processing & Analysis of Brain Imaging toolbox (DPABI; Yan, Wang, Zuo, & Zang, 2016). Preprocessing steps included: (a) slice‐timing correction for inter‐leaved acquisitions (27th slice was used as reference); (b) head motion correction (no participant had a head motion of more than 2 mm maximum displacement in any direction or 2° of any angular motion throughout the scan); (c) coregister of the structural image with the functional image; (d) new segmentation to DARTEL; (e) removal of nuisance variance through linear regression: six parameters from the head motion correction, white matter signal, cerebrospinal fluid signal, and global mean signal; (f) spatial normalization to the MNI (3 mm^3^); (g) spatial smoothing (FWHM = 4 mm); (h) removal of the linear trend in the time‐series; (i) band‐pass temporal filtering (0.01–0.1); and (j) extraction of the mean time courses of all the voxels in each seed region with the rest of the brain. It should be noted that four left‐lateralized participants (one musician) were excluded from the analysis due to excessive head motion. As left and right pars triangularis seeds, we used the activation clusters from the verb generation task corresponding to the right pars triangularis in right‐lateralized participants, and the left pars triangularis in left‐lateralized participants (see Figure S3; in the case of left‐lateralized participants, the voxel‐wise threshold was lowered until the local maxima located at the left pars triangularis formed its own independent cluster). Finally, the mean time courses of each seed were normalized to *z* values and contrasted between left‐lateralized and right‐lateralized participants using whole‐brain two‐sample *t*_*41*_ tests (voxel‐wise threshold of *p* < .001; FWE cluster‐corrected at *p* < .05), including musician/nonmusician as a dummy covariate of no interest.

## 3 RESULTS

### 3.1 Language lateralization of musicians versus nonmusicians

**Figure 1:**
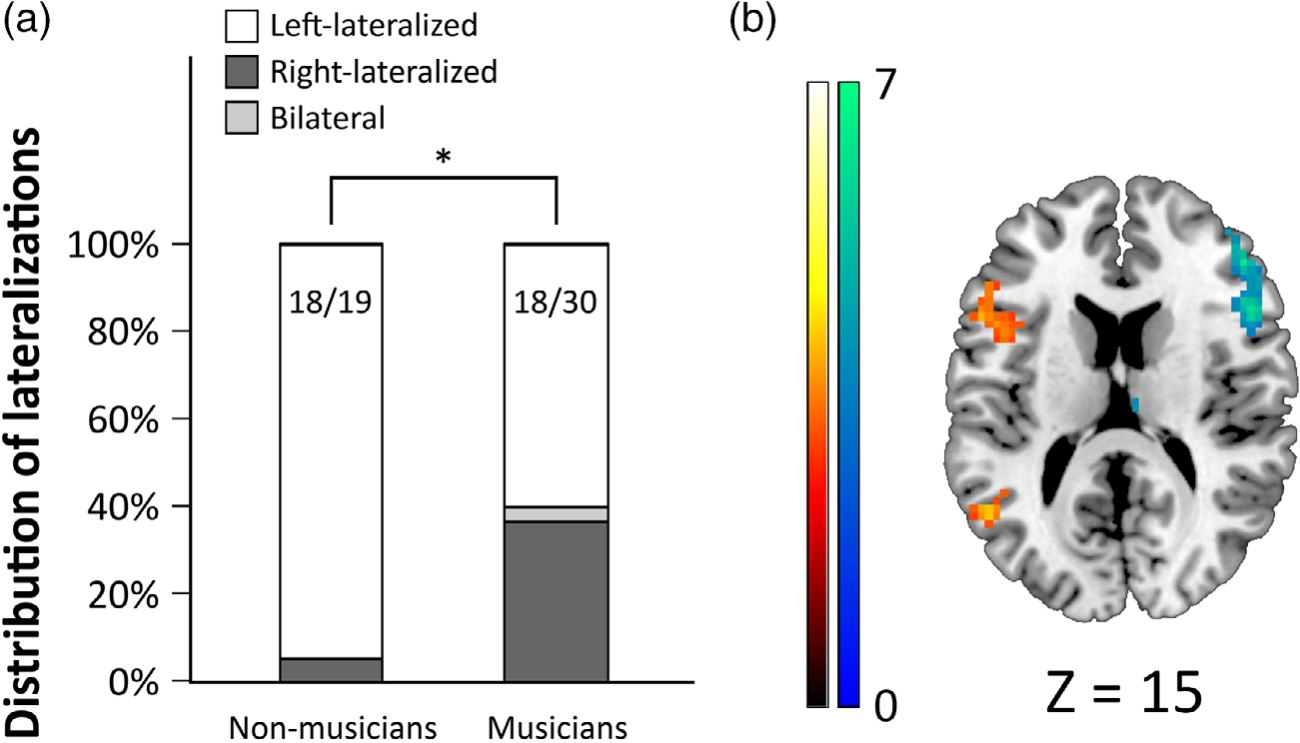
Language lateralization results. (a) Distribution of the different lateralization patterns derived from Laterality Indexes of musicians and nonmusicians. Musicians present a significantly higher incidence of atypical patterns (right‐lateralized and bilateral). *, Fisher's exact test for homogeneous distribution of atypical patterns of language lateralization; *p* = .008. (b) Differences in brain activity during the verb generation task according to language lateralization. *Left‐lateralized participants > right‐lateralized participants* (hot colors); and *right‐lateralized participants > left‐lateralized participants* (cold colors). Voxel‐wise threshold at *p* < .001, FWE cluster‐corrected at *p* < .05, coordinates reported in MNI space, colors bar represent *t* values. A full report of these results can be found in Figure S4 and Table S3

Language lateralization was assessed via the calculation of the LI (Laterality Index) for the verb generation task for each participant. Distributions of lateralization and LI across musicians and nonmusicians can be found in Figure 1 and Figure S2, respectively. A full report on the voxel data used for LI calculation, as well as the exact LI values for each participant, can be found in Table S1. Functional analysis of the verb generation task according to the language lateralization classification can be found in the supporting information. The language dominance distribution was as follows: only one nonmusician out of 19 (5.3% of nonmusicians) showed right‐lateralized language; 11 musicians of 30 (36.7% of musicians) showed right‐lateralized language; and 1 musician of 30 (3.3% of musicians) did not manifest a clear pattern of lateralization resulting from a LI of 5.97. Therefore, atypical patterns of language lateralization were found in 5.3% of nonmusicians, and in 40% of musicians. This difference in frequency of atypical lateralizations was statistically significant (Fisher's exact test; *p* = .008), with musicians presenting an incidence more than seven times greater than nonmusicians (risk ratio = 7.6; confidence interval = 1.07 to 53.82).

When analyzing left‐lateralized and right‐lateralized musicians, they did not significantly differ in the age of onset of musical training (*U* = 78; *p* = .289; left‐lateralized median = 6 years old; right‐lateralized median = 9 years old) or the amount of musical practice (*t*_*27*_ = 1.91; *p* = .067; left‐lateralized mean ± *SD* = 9,391.5 ± 3,134.9 hr; right‐lateralized mean ± *SD* = 7,194.7 ± 2,775.4 hr).

### 3.2 Gray matter volume and surface in left‐lateralized versus right‐lateralized

Following both our functional results and reports from previous studies, we analyzed gray matter structural differences between the left‐lateralized and right‐lateralized groups in: gray matter volume of the right HG, gyrification of the left HG, and cortical thickness of the right pars triangularis.

**Figure 2:**
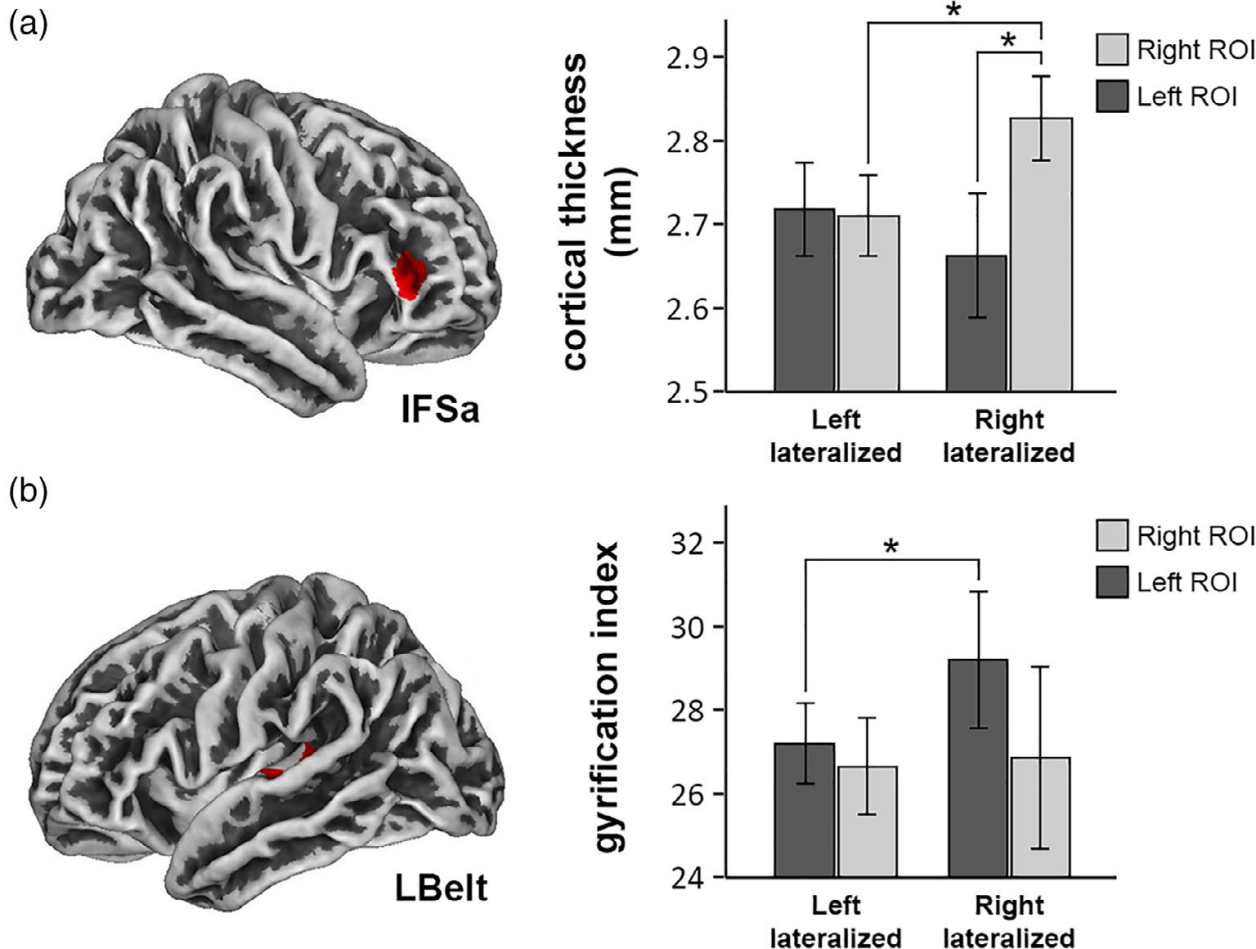
Results of the surface ROI analyses. (a) Cortical thickness of the IFSa area (HCP‐MMP1 surface atlas). (b) Gyrification index of the LBelt area (HCP‐MMP1 surface atlas). In accordance with our hypotheses, right‐lateralized participants presented both greater cortical thickness in the right IFSa (Mean ± *SD* of right‐lateralized vs. left‐lateralized = 2.83 ± 0.08 vs. 2.71 ± 0.14) and a higher gyrification index in the left LBelt (Mean ± *SD* of right‐lateralized vs. left‐lateralized = 29.21 ± 2.57 vs. 27.22 ± 2.87) than left‐lateralized participants. Asymmetry analyses also revealed a significant rightward hemispheric asymmetry of the IFSa in right‐lateralized participants (Mean ± *SD* of the left IFSa = 2.66 ± 0.12), whereas no asymmetry was found in left‐lateralized participants (Mean ± *SD* of the left IFSa = 2.72 ± 0.16). Error bars represent 95% confidence intervals. Asterisks represent statistical significance at *p* < .05

We did not find significant differences in the gray matter volume of the right HG. Surface analyses, however, confirmed our hypotheses about cortical thickness and gyrification (see Figure 2). Right‐lateralized participants presented a thicker cortex in the right pars triangularis (*p* = .021; FDR‐corrected) and a higher gyrification index in the lateral belt portion of the left HG (*p* = .017; FDR‐corrected). No significant differences were found in the gyrification index of the core and medial belt portions of the left HG. To further rule out the possible role of musicianship in these results, we performed the same analysis only for musicians, and the results were quite similar.

When exploring the hemispheric asymmetry of the aforementioned structural measures, no significant differences between the left‐lateralized and right‐lateralized groups were found in the AI of the gyrification of A1 (Mean ± *SD* of left‐lateralized vs. right‐lateralized = 0.005 ± 0.098 vs. –0.035 ± .086; *p* = .392), MBelt (−0.018 ± 0.09 vs. 0.009 ± 0.105; *p* = .234) or LBelt (0.012 ± 0.082 vs. 0.043 ± 0.065; *p* = .133), or in the AI of the gray matter volume of HG (0.061 ± 0.063 vs. 0.057 ± 0.077; *p* = .8). Cortical thickness of the IFSa region did, however, significantly differ in its AI when comparing the two groups (0.001 ± 0.033 vs. –0.03 ± 0.022; *p* = .012, FDR‐corrected). Further analyses confirmed that, whereas the left‐lateralized group presented no hemispheric asymmetry in the cortical thickness of this region (*p* = .532), the right‐lateralized group showed a rightward asymmetry (*p* = .001, FDR‐corrected; see Figure 2). In fact, all the right‐lateralized participants, except one (who showed an AI of 0.002), presented a rightward AI in the cortical thickness of IFSa.

### 3.3 Functional connectivity differences in left‐lateralized versus right‐lateralized

Finally, we explored the functional connectivity at rest (rs‐FC) of the task‐relevant pars triangularis of the IFG in both groups. Thus, we performed whole‐brain two‐sample *t*_*41*_ tests (voxel‐wise threshold of *p* < .001; FWE cluster‐corrected at *p* < .05) left‐lateralized and right‐lateralized participants, comparing the rs‐FC of their left pars triangularis and right pars triangularis.

**Figure 3:**
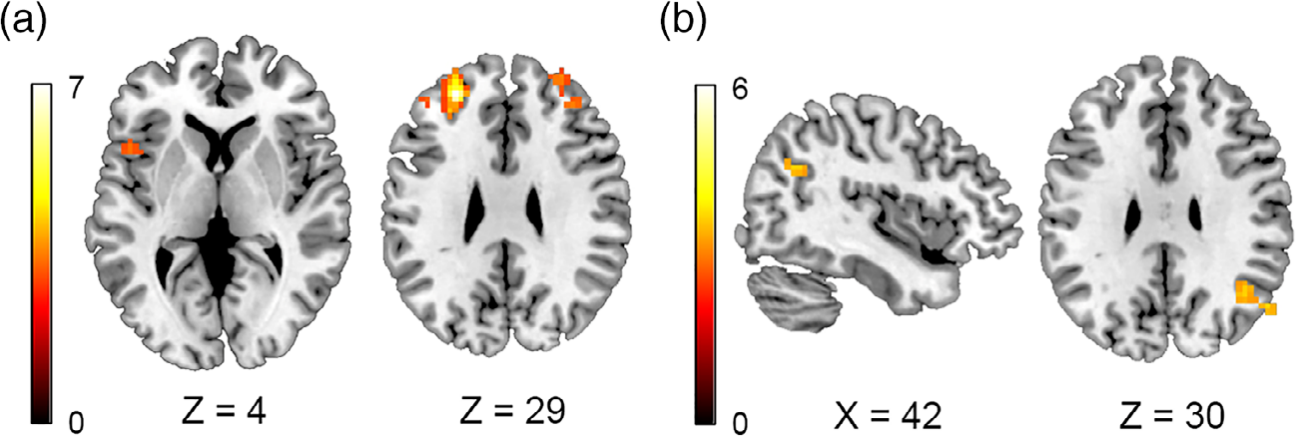
Results of the seed‐based resting‐state functional connectivity analysis (voxel‐wise threshold at *p* < .001, FWE cluster‐corrected at *p* < .05, coordinates reported in MNI space, color bars represent *t* values). (a) Right pars triangularis seed: *left‐lateralized > right‐lateralized*. (b) Right pars triangularis seed: *right‐lateralized > left‐lateralized*. No statistically significant differences were found when exploring the left pars triangularis seed

Results for each seed region can be found in Figure 3 and Table S4. Statistically significant differences at the pre‐established threshold were only found in the rs‐FC of the right pars triangularis. Left‐lateralized participants showed positive rs‐FC with part of the left Broca's area/left anterior insula, as well as the middle frontal gyrus of both hemispheres. Right‐lateralized participants, however, showed a negative rs‐FC between their right pars triangularis and these regions. On the other hand, right‐lateralized participants presented a positive rs‐FC with the right angular gyrus (coincident with the right angular gyrus that was significantly more active during the task), right cerebellum lobule IX, and bilateral lingual gyrus, in contrast to the negative rs‐FC found in the left‐lateralized group. To further rule out the possible role of musicianship in these results, we performed the same analysis only for musicians, and the results were quite similar.

## 4 DISCUSSION

Our results showed that atypical patterns of language processing (right‐lateralized and bilateral) were significantly more prevalent in musicians (40%) than in nonmusicians (5.3%), suggesting that a common factor links musicianship and atypical dominance of language in the brain of left‐handers. Further analysis revealed that atypical language dominance was associated with greater cortical thickness in the right pars triangularis of the IFG, as well as a rightward hemispheric asymmetry in this region and a higher gyrification index in the lateral belt portion of the left HG. Functionally, the right pars triangularis of individuals with atypical language dominance was more coupled at rest with the right angular gyrus, and less coupled with the left IFG. Overall, we suggest that atypical language dominance and musicianship may both arise from specific anatomic and functional characteristics of the brain that enhance the role of the right IFG and right HG.

Overall, the incidence of atypical dominance for language in our study is 26%, which is similar to previous reports in non‐right‐handed populations (Mazoyer et al., 2014; Pujol et al., 1999; Szaflarski et al., 2002). However, compared to these reports, our study presents differences in the composition of this atypical lateralization group: we found a lower incidence of bilateral cases (2% vs. 14–15%) and a higher incidence of right‐lateralized cases (24% vs. 7–10%). Nevertheless, our main finding is that atypical dominance was found more frequently in left‐handed musicians (40%) than in left‐handed nonmusicians (5.3%). If we focus only on right‐lateralized cases, the differences are evident: right dominance of language was present in 37% of our left‐handed musicians, in contrast with the 7–10% previously described in the non‐right‐handed population. Taking into account the low incidence found among nonmusicians (5.3%), the absence of training‐related differences between left‐ and right‐dominant musicians, and most importantly, the lack of support for sensory influences in determining language lateralization (Van der Haegen et al., 2016), it can be tentatively suggested that some of the brain factors favoring an atypical organization may also predispose left‐handers to musicianship. That is, rather than an effect of musical training on hemispheric language dominance development, we may have found evidence of a common facilitator factor for both. Our results have also determined which structural and functional factors may lie behind this facilitation. Structural analyses revealed that some of these factors were a thicker cortex in the right pars triangularis of the IFG, along with a rightward hemispheric asymmetry of this region's thickness (vs. the symmetric pattern found in the typically lateralized group). These results are consistent with previous results in Wada‐tested patients (Foundas et al., 1996; Keller et al., 2018) and fMRI‐assessed healthy participants (Josse et al., 2009) that have highlighted the rightward structural asymmetry of the IFG as a marker of atypical language dominance. Importantly, musicians also presented greater cortical thickness in the right pars triangularis (Bermudez et al., 2009), and this thicknesss correlated positively with pitch discrimination ability in nonmusicians (Novén et al., 2018). It should be noted that neuroanatomical correlates of musicianship are thought to stem from both experiential factors (musical training) and preexisting factors (Foster & Zatorre, 2010; Vaquero et al., 2018). Hence, greater cortical thickness of the right pars triangularis, resulting in a rightward hemispheric asymmetry, could be acting as a predisposing factor to both musicianship and atypical language dominance. A second predisposing structural factor is the higher gyrification index in the lateral belt portion of the left HG, which is consistent with previous data showing that left HG gyri duplications are related to a loss of leftward lateralization during receptive language processing (Tzourio‐Mazoyer et al., 2015). Similarly, gyrification in the HG was also associated with musicianship (Benner et al., 2017) and pitch perception preference (Schneider et al., 2005). It should be noted that gyrification in the auditory cortex is grossly established prenatally (Chi, Dooling, & Gilles, 1977; Hill et al., 2010), showing relatively little change during the first years of life, compared to other brain cortices (Li et al., 2014). Hence, the early development of HG gyrification is consistent with its possible facilitator role in both atypical language dominance and musicianship. Another factor we expected to find was greater gray matter volume in the right HG of right‐lateralized participants, which would be consistent with the proposal of superior temporal gyrus asymmetry as the basis for language dominance (Geschwind & Levitsky, 1968) and parallel previous findings in musicians (Bermudez et al., 2009; Palomar‐García et al., 2017). However, we did not find any differences between left‐handers with typical and atypical language dominance.

Functional factors were found by exploring the brain activations during the language task and the rs‐FC of the task‐relevant pars triangularis of the IFG. Atypical language dominance was associated with higher activation of the right IFG, right angular gyrus, right thalamus, and left cerebellum. Especially noteworthy is the prominent role of the right pulvinar nucleus in atypical participants, whose left counterpart has been previously revealed to present anatomic connections with the left pars triangularis in right‐handers, having been suggested as a basis for lexical‐semantic processing during word production (Bohsali et al., 2015). This brain activity pattern corresponds to the one previously described in healthy left‐handers presenting the most negative LI values (Zago et al., 2017), and it suggests that atypical language dominance is supported by greater participation during language processing of a set of distributed language‐related regions of the right hemisphere; that is, language control during the task is hemispherically reversed. Consistent with task activations, the participants with atypical dominance for language showed more rs‐FC between the right pars triangularis of the IFG and the right angular gyrus, as well as less rs‐FC with part of the left Broca's area/insula. There were no differences between the two groups in rs‐FC of the left pars triangularis of the IFG. This pattern (and also the one extracted from the task) means that the key functional components facilitating atypical dominance for language are a strong rs‐FC within the language areas of the right hemisphere and a weak interhemispheric rs‐FC. This matches a previous proposal from transversal (Friederici et al., 2011) and longitudinal (Szaflarski et al., 2006) studies that typical language dominance development is accompanied by an increase in rs‐FC within left language areas and a decrease in interhemispheric rs‐FC (Tzourio‐Mazoyer, Perrone‐Bertolotti, Jobard, Mazoyer, & Baciu, 2017). It is also consistent with the hypothesis that top‐down influences during language processing from anterior language areas are responsible for the lateralization of posterior language areas, such as the visual word form area (Cai, Lavidor, Brysbaert, Paulignan, & Nazir, 2008; Cai, Paulignan, Brysbaert, Ibarrola, & Nazir, 2010). But most interestingly, musicianship has also been associated with an increased rs‐FC between right auditory and right motor areas, as well as a decreased interhemispheric rs‐FC in motor areas (Palomar‐García et al., 2017). Thus, we suggest that the differential development of the right inter‐ and intra‐hemispheric connections is a functional factor that facilitates both atypical language dominance and musicianship.

Previous studies have already shown interactions between language function and musicianship, both behaviorally (Slater & Kraus, 2016) and neurally (Musacchia et al., 2007). Hence, in light of the present results, it is plausible to suggest that the development of language lateralization and musicianship shares some cerebral factors. In fact, considering left‐handedness as an indicative factor of a possible underlying atypical dominance for language, some studies have reported a higher proportion of left‐handers among musical students (Götestam, 1990) and professional musicians (Aggleton, Kentridge, & Good, 1994) than in the general population, as well as greater proficiency in left‐handed musicians than in right‐handed musicians when confronted with difficult musical tasks (Kopiez, Galley, & Lee, 2006; Smit & Sadakata, 2018). These findings would hint at a relationship between musical aptitude and atypical language dominance. Suggesting this kind of developmental relationship between language and musicianship might sound too audacious because we are directly relating the musician/nonmusician status to innate musical aptitude (plus its related cerebral characteristics), thus taking for granted that all the participants in our study had equal chances of initiating and completing official musical training. However, we would like to highlight the unique musical tradition and culture of the region where this study took place: the Valencian Community (Rodrigo‐Mancho, 1993). This region comprises 9.4% of the total population of Spain, and yet it makes up 50% of the total number of musicians residing in this country, averaging 3.1 federated musical associations per town, which is nine times greater than the national average (FSMCV, 2015). As a result, access to musical training is widely offered and fomented in this region. Hence, within this environment, we think our assumption would be correct, and the distinction between musicians and nonmusicians should be very closely related to innate musical aptitude.

## 5 LIMITATIONS AND FUTURE STUDIES

This study has some limitations. Most importantly, the sample sizes are small, which could have led to an overestimation of the reported effect sizes (Brysbaert, 2019). This is of particular concern in the case of the nonmusician group, which is the less represented group. Hence, caution should be used when drawing conclusions based on these results. Future studies should address this issue by replicating the effects in larger sample sizes.

We could not explore the bilateral pattern of language lateralization because our classification was able to detect only one such participant. Additionally, our classification as “atypically lateralized” was broader than those used in some other studies (Mazoyer et al., 2014; Zago et al., 2017), which supports the need for a more fine‐grained division of atypical lateralization. Hence, future studies might be interested in exploring the reported results in relation to the different phenotypes present in atypical lateralization (for a review, see Vingerhoets, 2019 and its comments).

## 6 CONCLUSION

We found a higher incidence of atypical language dominance in left‐handed musicians compared to left‐handed nonmusicians. We also presented structural and functional factors that could act as facilitators for both atypical language dominance and musicianship: (a) greater cortical thickness in the right pars triangularis of the IFG, concurrently with a rightward hemispheric asymmetry of this region's thickness; (b) a higher gyrification index in the left HG; and (c) stronger rs‐FC between the anterior and posterior task‐relevant language regions of the right hemisphere and weaker interhemispheric FC between anterior regions. We conclude that atypical language dominance and musicianship might share common developmental bases in the brains of left‐handers.

## CONFLICT OF INTEREST

The authors declare no conflict of interests.

## Supporting information


**Appendix** **S1**: Supporting Information

**Supplementary Figure 1** Inclusive mask used in the calculation of the Laterality Index. Coordinates are reported in the MNI space.

**Supplementary Figure 2** Laterality Indexes report. Distribution of Laterality Index (LI), represented in intervals of 20, among musicians and nonmusicians. Distribution of LI in musicians is more skewed towards the right‐lateralizations (nonmusicians LI mean ± SD = 77.97 ± 43.23; musicians LI mean ± SD = 27.1 **±** 86.9).

**Supplementary Figure 3** Brain activation maps resulting from the *activation > control* contrast during the verb generation task. Voxel‐wise threshold at *p* < 0.001, FWE cluster‐corrected at *p* < 0.05, coordinates reported in MNI space, color bars represent *t* values. (**A**) Left‐lateralized group. (**B**) Right‐lateralized group. Between‐groups parallelism of task‐related activations is not only evident in the inferior frontal area, but also in the cerebelum, which supports our lateralization assessment. It should be noted that, when using a less strict threshold in the right‐lateralized group (voxel‐wise threshold of *p* < 0.005; FWE corrected at *p* < 0.05), insula activity was also found in the left hemisphere, thus matching the pattern observed in the left‐lateralized group, and pointing to the pars triangularis cluster as the truly lateralized frontal activity. Activation clusters comprising the pars triangularis of the IFG (right for right‐lateralizeds, left for left‐lateralizeds) were used as seeds for the resting‐state functional connectivity analyses.

**Supplementary Figure 4** Differences in brain activity during the verb generation task according to language lateralization. Voxel‐wise threshold at *p* < 0.001, FWE cluster‐corrected at *p* < 0.05, coordinates reported in MNI space, color bars represent *t* values. *Left‐lateralized participants > right‐lateralized participants* (hot colors); and *right‐lateralized participants > left‐lateralized participants* (cold colors). Note that there are no differences in the anterior insula regions depicted in the one‐samples, thus confirming that anterior lateralization differences are confined to the pars triangularis.

**Supplementary Table 1** Data used in the calculation of Laterality Indexes (LI). Note that unsmoothed images were used. ID = identification; M = musician; NM = non‐musician; *k* = voxel count (ROI); *t* = peak *t* value (ROI).

**Supplementary Table 2** Cerebral activations of left‐lateralized and right‐lateralized groups during the verb generation task. Voxel‐wise threshold at *p* < 0.001, FWE cluster‐corrected at *p* < 0.05, coordinates reported in the MNI space. L = left, R = right.

**Supplementary Table 3** Differences between left‐lateralized and right‐lateralized groups in brain activity during the verb generation task. Voxel‐wise threshold at *p* < 0.001, FWE cluster‐corrected at *p* < 0.05, coordinates reported in the MNI space. L = left, R = right.

**Supplementary Table 4** Differences between left‐lateralized and right‐lateralized groups in seed‐based resting‐state functional connectivity. Voxel‐wise threshold at *p* < 0.001, FWE cluster‐corrected at *p* < 0.05, coordinates reported in the MNI space. L = left, R = right.

## Data Availability

The data that support the findings of this study are available from the corresponding author upon reasonable request.
