# Supplementary material for: Left‐handed musicians show a higher probability of atypical cerebral dominance for language
Source: Hum Brain Mapp. 2020 Feb 7;41(8):2048–58. doi: 10.1002/hbm.24929 (PMC7268010; doi:10.1002/hbm.24929)
Supplement: Supplementary file 1 — Appendix S1: Supporting Information [file HBM-41-2048-s001.docx]

**SUPPLEMENTARY MATERIAL**

*Verb generation task in left-lateralized vs. right-lateralized*

Whole-brain one-sample *t* tests (voxel-wise threshold of *p* < 0.001; FWE cluster-corrected at *p* < 0.05) for the verb generation task of left-lateralized (d.f. = 34) and right-lateralized (d.f. = 10) groups can be found in Supplementary Fig. 3 and Supplementary Table 2. Brain activity clusters in both groups mainly consisted of: (1) bilateral anterior insula; (2) lateralized inferior frontal gyrus/pars triangularis; (3) supplementary motor area; and (4) contralaterally lateralized cerebelum crus 1/2.

Whole-brain two-sample *t_45_* tests (voxel-wise threshold of *p* < 0.001; FWE cluster-corrected at *p* < 0.05) for the verb generation task between left-lateralized and right-lateralized groups can be found in Supplementary Fig. 4 and Supplementary Table 3. These results confirm that the two groups do not significantly differ in the brain activity of the anterior insula clusters previously described in the one-sample *t* tests, but rather in the inferior frontal gyrus/pars triangularis cluster. The activity of this region is found to be higher in the left hemisphere of the left-lateralized group and in the right hemisphere of the right-lateralized group. Left-lateralized participants also present higher activation in the left precentral gyrus and the left angular gyrus. On the other hand, right-lateralized participants show higher activation in the right dorsomedial/pulvinar nuclei of the thalamus, left cerebelum crus 1, right inferior temporal gyrus, and right angular gyrus. To further rule out the possible role of musicianship in these results, we performed the same analysis only for musicians, and the results were quite similar.


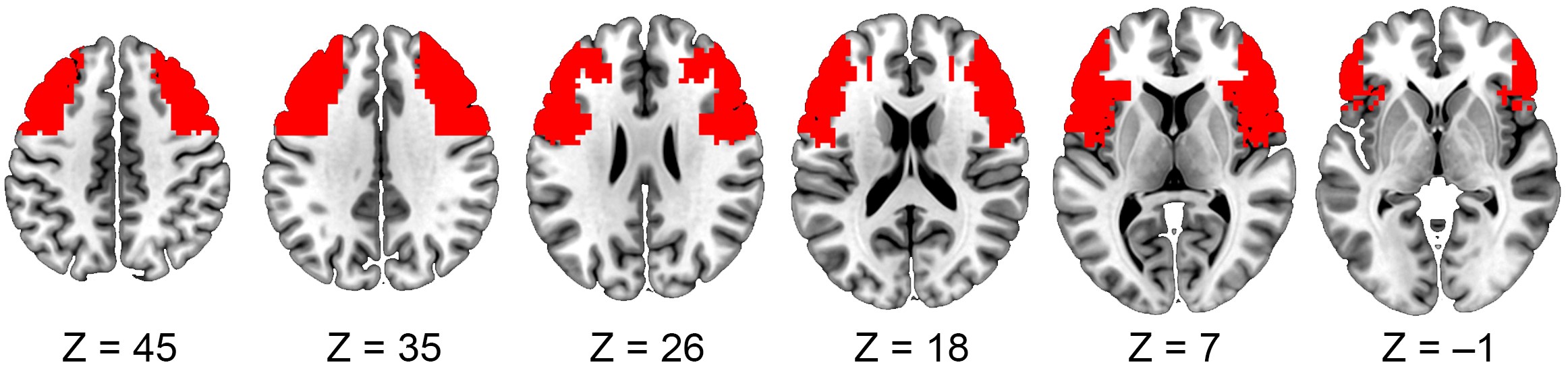


**Supplementary Figure 1**. Inclusive mask used in the calculation of the Laterality Index. Coordinates are reported in the MNI space.

**
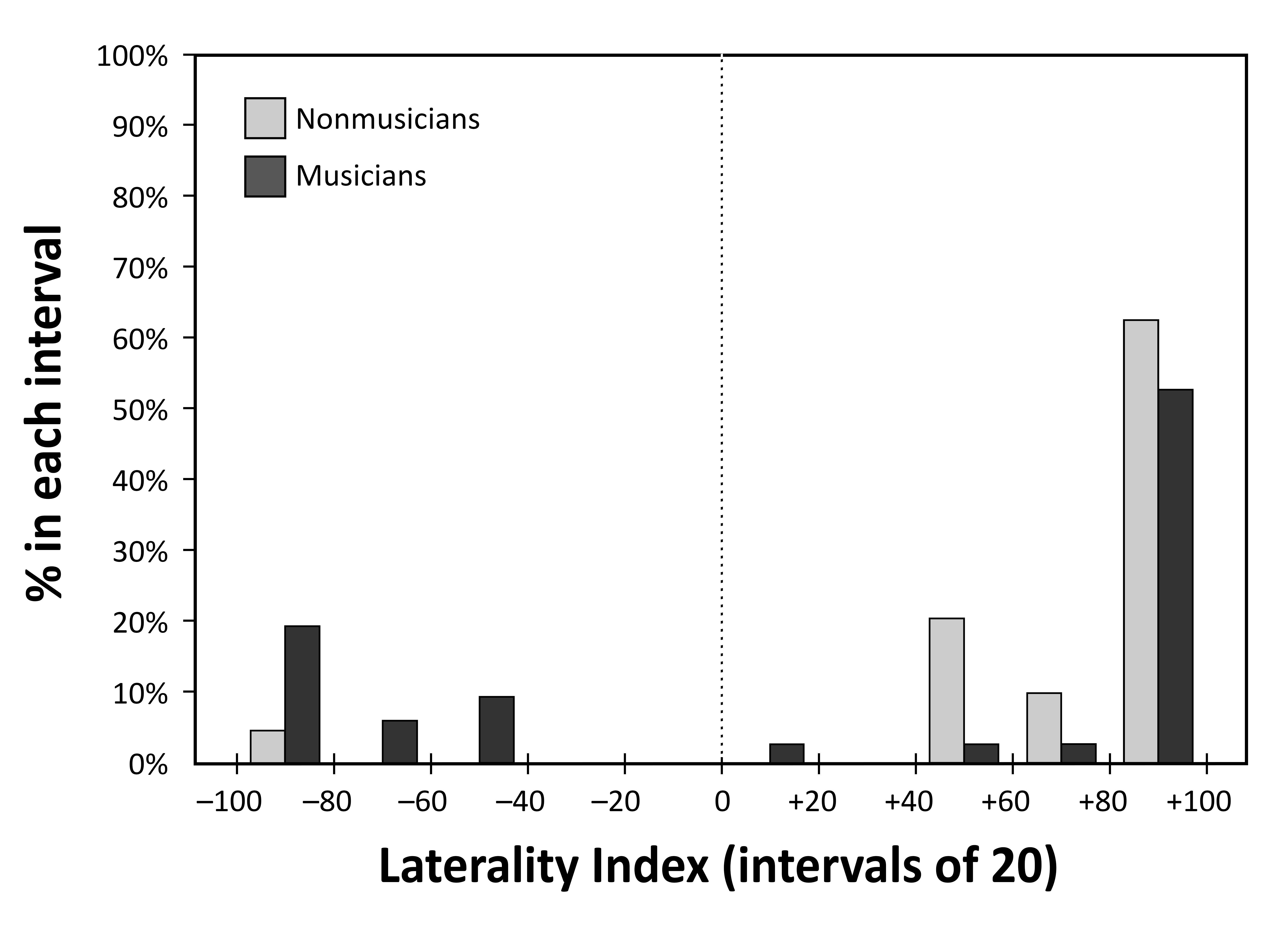
**

**Supplementary Figure 2**. Laterality Indexes report. Distribution of Laterality Index (LI), represented in intervals of 20, among musicians and non-musicians. Distribution of LI in musicians is more skewed towards the right-lateralizations (non-musicians LI mean ± SD = 77.97 ± 43.23; musicians LI mean ± SD = 27.1 **±** 86.9).


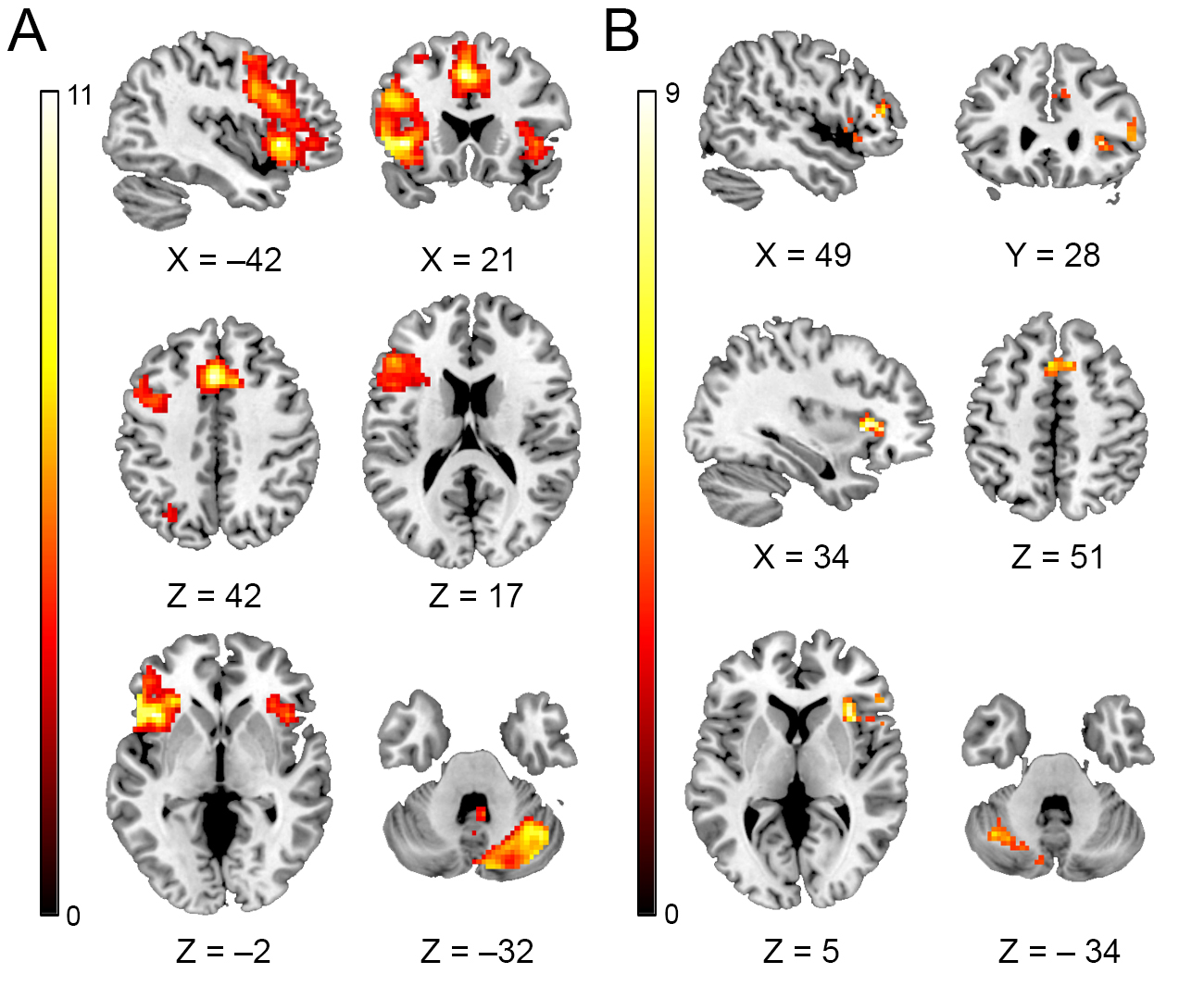


**Supplementary Figure 3.** Brain activation maps resulting from the *activation > control* contrast during the verb generation task. Voxel-wise threshold at *p* < 0.001, FWE cluster-corrected at *p* < 0.05, coordinates reported in MNI space, color bars represent *t* values. (**A**) Left-lateralized group. (**B**) Right-lateralized group. Between-groups parallelism of task-related activations is not only evident in the inferior frontal area, but also in the cerebelum, which supports our lateralization assessment. It should be noted that, when using a less strict threshold in the right-lateralized group (voxel-wise threshold of *p* < 0.005; FWE corrected at *p* < 0.05), insula activity was also found in the left hemisphere, thus matching the pattern observed in the left-lateralized group, and pointing to the pars triangularis cluster as the truly lateralized frontal activity. Activation clusters comprising the pars triangularis of the IFG (right for right-lateralizeds, left for left-lateralizeds) were used as seeds for the resting-state functional connectivity analyses.


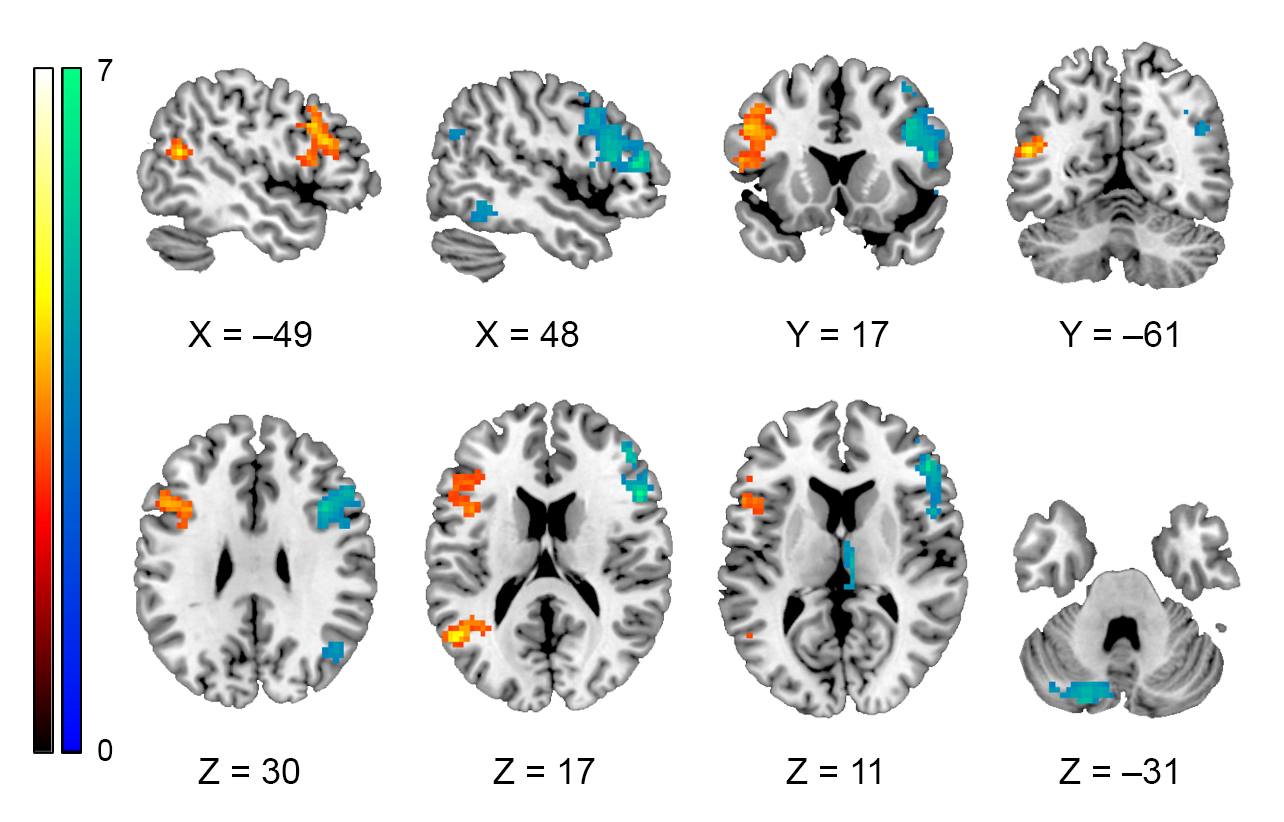


**Supplementary Figure 4**. Differences in brain activity during the verb generation task according to language lateralization. Voxel-wise threshold at *p* < 0.001, FWE cluster-corrected at *p* < 0.05, coordinates reported in MNI space, color bars represent *t* values. *Left-lateralized participants > right-lateralized participants* (hot colors); and *right-lateralized participants > left-lateralized participants* (cold colors). Note that there are no differences in the anterior insula regions depicted in the one-samples, thus confirming that anterior lateralization differences are confined to the pars triangularis.

| ID | group | Left hemisphere | | Right hemisphere | | LI |
| --- | --- | --- | --- | --- | --- | --- |
|  |  | *k* | *t* | *k* | *t* |  |
|  |  |  |  |  |  |  |
| 1 | NM | 306 | 15.29 | - | - | 100 |
| 2 | M | 371 | 12.02 | - | - | 100 |
| 3 | M | 339 | 11.96 | - | - | 100 |
| 4 | NM | 186 | 8.45 | - | - | 100 |
| 5 | NM | 159 | 8.02 | - | - | 100 |
| 6 | M | 285 | 7.62 | - | - | 100 |
| 7 | NM | 138 | 7.45 | - | - | 100 |
| 8 | M | 186 | 7.42 | - | - | 100 |
| 9 | NM | 38 | 7.16 | - | - | 100 |
| 10 | M | 88 | 7.11 | - | - | 100 |
| 11 | M | 25 | 6.89 | - | - | 100 |
| 12 | M | 135 | 6.82 | - | - | 100 |
| 13 | M | 247 | 6.63 | - | - | 100 |
| 14 | NM | 140 | 6.6 | - | - | 100 |
| 15 | NM | 311 | 6.21 | - | - | 100 |
| 16 | NM | 149 | 6.01 | - | - | 100 |
| 17 | M | 221 | 5.85 | - | - | 100 |
| 18 | NM | 18 | 5.72 | - | - | 100 |
| 19 | M | 117 | 5.54 | - | - | 100 |
| 20 | NM | 13 | 5.05 | - | - | 100 |
| 21 | NM | 39 | 4.62 | - | - | 100 |
| 22 | M | 70 | 3.8 | - | - | 100 |
| 23 | M | 68 | 3.66 | - | - | 100 |
| 24 | NM | 74 | 4.96 | - | - | 100 |
| 25 | M | 22 | 3.75 | - | - | 100 |
| 26 | M | 184 | 8.38 | 14 | 5.02 | 85.9 |
| 27 | NM | 423 | 9.76 | 33 | 6.06 | 85.5 |
| 28 | M | 394 | 7.21 | 35 | 5.29 | 83.7 |
| 29 | NM | 231 | 9.74 | 28 | 5.72 | 78.4 |
| 30 | NM | 285 | 7.94 | 36 | 5.98 | 77.6 |
| 31 | M | 160 | 6.14 | 23 | 3.9 | 74.9 |
| 32 | NM | 434 | 7.04 | 115 | 7.05 | 58.1 |
| 33 | M | 178 | 8.89 | 50 | 4.83 | 56.1 |
| 34 | NM | 170 | 6.54 | 48 | 5.23 | 56 |
| 35 | NM | 436 | 8.63 | 125 | 5.56 | 55.4 |
| 36 | NM | 290 | 6.43 | 85 | 5.58 | 54.7 |
| 37 | M | 417 | 8.66 | 370 | 8.9 | 6 |
| 38 | M | 135 | 5.34 | 376 | 5.86 | –47.2 |
| 39 | M | 74 | 4.54 | 229 | 5.52 | –51.2 |
| 40 | M | 98 | 6.8 | 379 | 9.87 | –58.9 |
| 41 | M | 67 | 4.46 | 304 | 4.69 | –63.9 |
| 42 | M | 21 | 4.66 | 132 | 5.61 | –72.5 |
| 43 | NM | 28 | 5.07 | 327 | 8.52 | –84.2 |
| 44 | M | - | - | 29 | 5.09 | –100 |
| 45 | M | - | - | 14 | 5.4 | –100 |
| 46 | M | - | - | 10 | 5.19 | –100 |
| 47 | M | - | - | 3 | 5.28 | –100 |
| 48 | M | - | - | 15 | 5.76 | –100 |
| 49 | M | - | - | 281 | 6.26 | –100 |
|  |  |  |  |  |  |  |

**Supplementary Table 1**. Data used in the calculation of Laterality Indexes (LI). Note that unsmoothed images were used. ID = identification; M = musician; NM = non-musician; *k* = voxel count (ROI); *t* = peak *t* value (ROI).

**Supplementary Table 2**. Cerebral activations of left-lateralized and right-lateralized groups during the verb generation task. Voxel-wise threshold at *p* < 0.001, FWE cluster-corrected at *p* < 0.05, coordinates reported in the MNI space. L = left, R = right.

| Region  (peak) | BA  (cluster) | *k* | X | Y | Z | *t*- value  (peak) |
| --- | --- | --- | --- | --- | --- | --- |
| *a) Left-lateralized* | | | | | | |
| R cerebelum crus 2 | - | 500 | 18 | −82 | −37 | 11.48 |
| L anterior insula | 6, 8, 32, 44, 45, 47, 48 | 1984 | −42 | 20 | −1 | 11.29 |
| R anterior insula | 47, 48 | 140 | 42 | 20 | −1 | 6.54 |
| R cerebelum vermis | - | 40 | 6 | −49 | −31 | 5.99 |
| L angular gyrus | 7 | 33 | −30 | −67 | 41 | 4.42 |
| *b) Right-lateralized* | | | | | | |
| R anterior insula | 47, 48 | 68 | 33 | 17 | 2 | 9.41 |
| R SMA | 6, 32 | 121 | 9 | 11 | 59 | 7.82 |
| L cerebelum vermis | - | 36 | 0 | −52 | −19 | 7.68 |
| L cerebelum crus 1 | - | 91 | −36 | −55 | −37 | 6.95 |
| R pars triangularis | 45 | 27 | 48 | 38 | 11 | 6.86 |

**Supplementary Table 3**. Differences between left-lateralized and right-lateralized groups in brain activity during the verb generation task. Voxel-wise threshold at *p* < 0.001, FWE cluster-corrected at *p* < 0.05, coordinates reported in the MNI space. L = left, R = right.

| Region  (peak) | BA  (cluster) | *k* | X | Y | Z | *t*- value  (peak) |
| --- | --- | --- | --- | --- | --- | --- |
| *a) Left-lateralized* > *Right-lateralized* | | | | | | |
| L middle frontal gyrus | 44, 45, 46, 48 | 245 | −42 | 20 | 41 | 7.2 |
| L angular gyrus | 21, 37, 39 | 63 | −39 | −52 | 20 | 5.64 |
| L precentral gyrus | 6, 8, 9 | 38 | −36 | −4 | 59 | 4.53 |
| *b) Right-lateralized* > *Left-lateralized* | | | | | | |
| R pars triangularis | 6, 8, 9, 44, 45, 48 | 457 | 51 | 20 | 17 | 6.6 |
| L cerebellum crus 1 | - | 77 | −15 | −76 | −28 | 5.14 |
| R thalamus | - | 31 | 6 | −19 | 11 | 4.74 |
| R inferior temporal gyrus | 20, 37 | 28 | 51 | −49 | −13 | 4.51 |
| R angular gyrus | 7, 19, 39, 40 | 58 | 36 | −73 | 35 | 4.1 |

**Supplementary Table 4**. Differences between left-lateralized and right-lateralized groups in seed-based resting-state functional connectivity. Voxel-wise threshold at *p* < 0.001, FWE cluster-corrected at *p* < 0.05, coordinates reported in the MNI space. L = left, R = right.

| Region  (peak) | BA  (cluster) | *k* | X | Y | Z | *t*- value  (peak) | | *z- value mean* ± SD  (cluster) |
| --- | --- | --- | --- | --- | --- | --- | --- | --- |
| *a) SEED: R pars triangularis / Left-lateralized* > *Right-lateralized* | | | | | | | |  |
| L middle frontal gyrus | 9, 45, 46 | 132 | −27 | 42 | 27 | | 7.1 | 0.04 ± 0.11 > −0.24 ± 0.09 |
| R middle frontal gyrus | 46 | 58 | 24 | 45 | 18 | | 4.98 | 0.12 ± 0.16 > −0.13 ± 0.12 |
| L anterior insula | 45, 48 | 51 | −42 | 12 | −3 | | 4.48 | 0.16 ± 0.16 > −0.1 ± 0.17 |
| *b) SEED: R pars triangularis / Right-lateralized* > *Left-lateralized* | | | | | | | |  |
| R cerebellum IX | - | 30 | 9 | −51 | −45 | | 5.55 | 0.04 ± 0.10 > −0.19 ± 0.10 |
| L lingual gyrus | 17, 30 | 89 | −6 | −57 | 6 | | 4.81 | 0.03 ± 0.15 > −0.24 ± 0.16 |
| R angular gyrus | 39 | 30 | 54 | −66 | 30 | | 3.82 | 0.11 ± 0.19 > −0.17 ± 0.18 |
