# Supplementary figures and images for: Left‐handed musicians show a higher probability of atypical cerebral dominance for language
Source: Hum Brain Mapp. 2020 Feb 7;41(8):2048–58. doi: 10.1002/hbm.24929 (PMC7268010; doi:10.1002/hbm.24929)

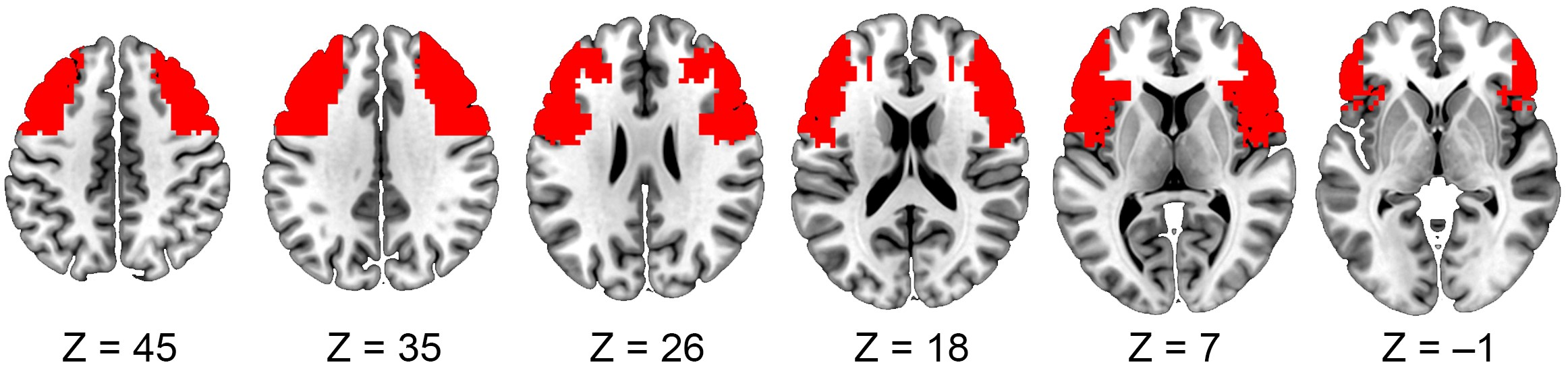

Supplement: Supplementary file 2 — Supplementary Figure 1 Inclusive mask used in the calculation of the Laterality Index. Coordinates are reported in the MNI space. [file HBM-41-2048-s002.tif]

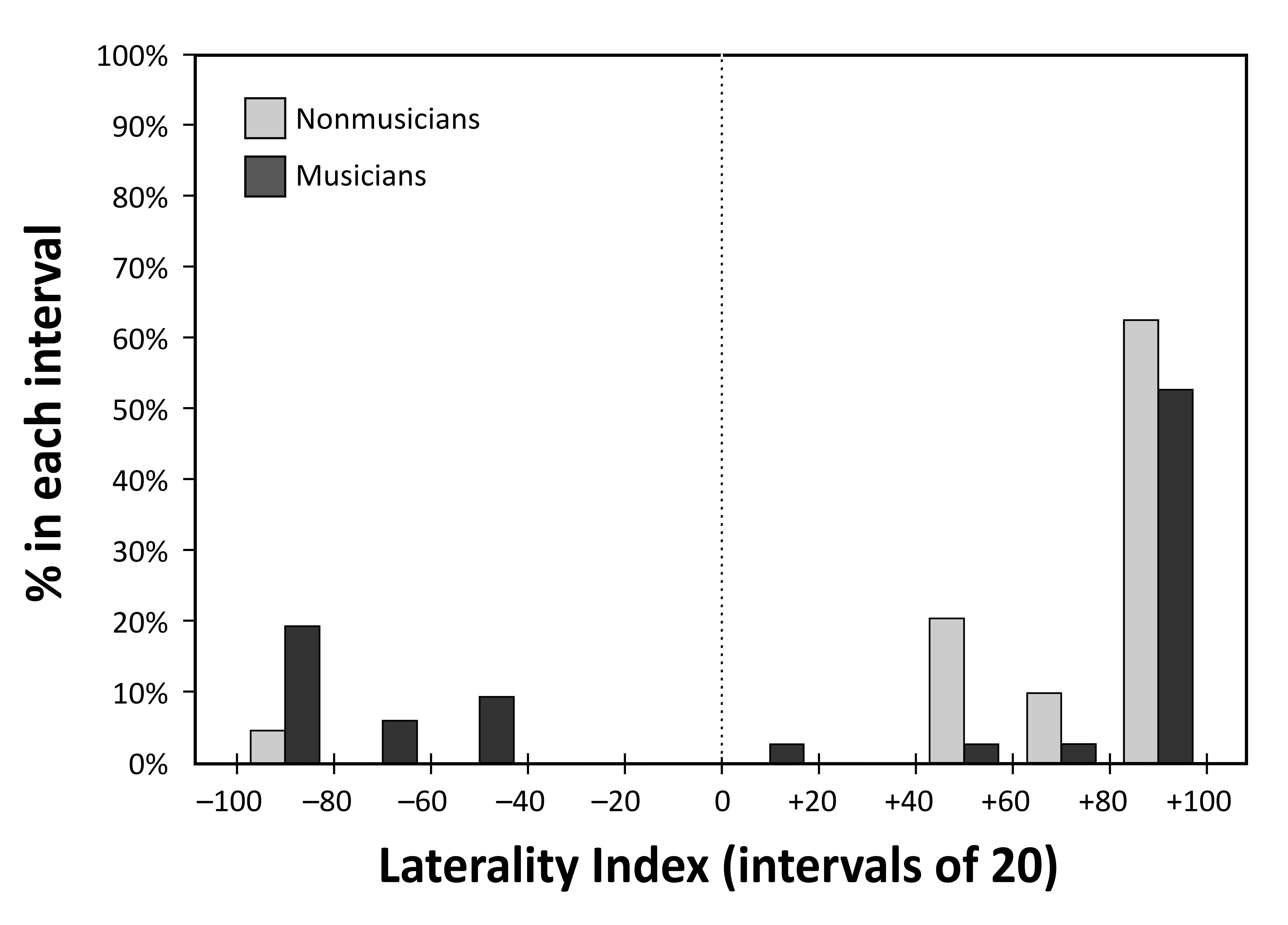

Supplement: Supplementary file 3 — Supplementary Figure 2 Laterality Indexes report. Distribution of Laterality Index (LI), represented in intervals of 20, among musicians and nonmusicians. Distribution of LI in musicians is more skewed towards the right‐lateralizations (nonmusicians LI mean ± SD = 77.97 ± 43.23; musicians LI mean ± SD = 27.1 ± 86.9). [file HBM-41-2048-s003.tif]

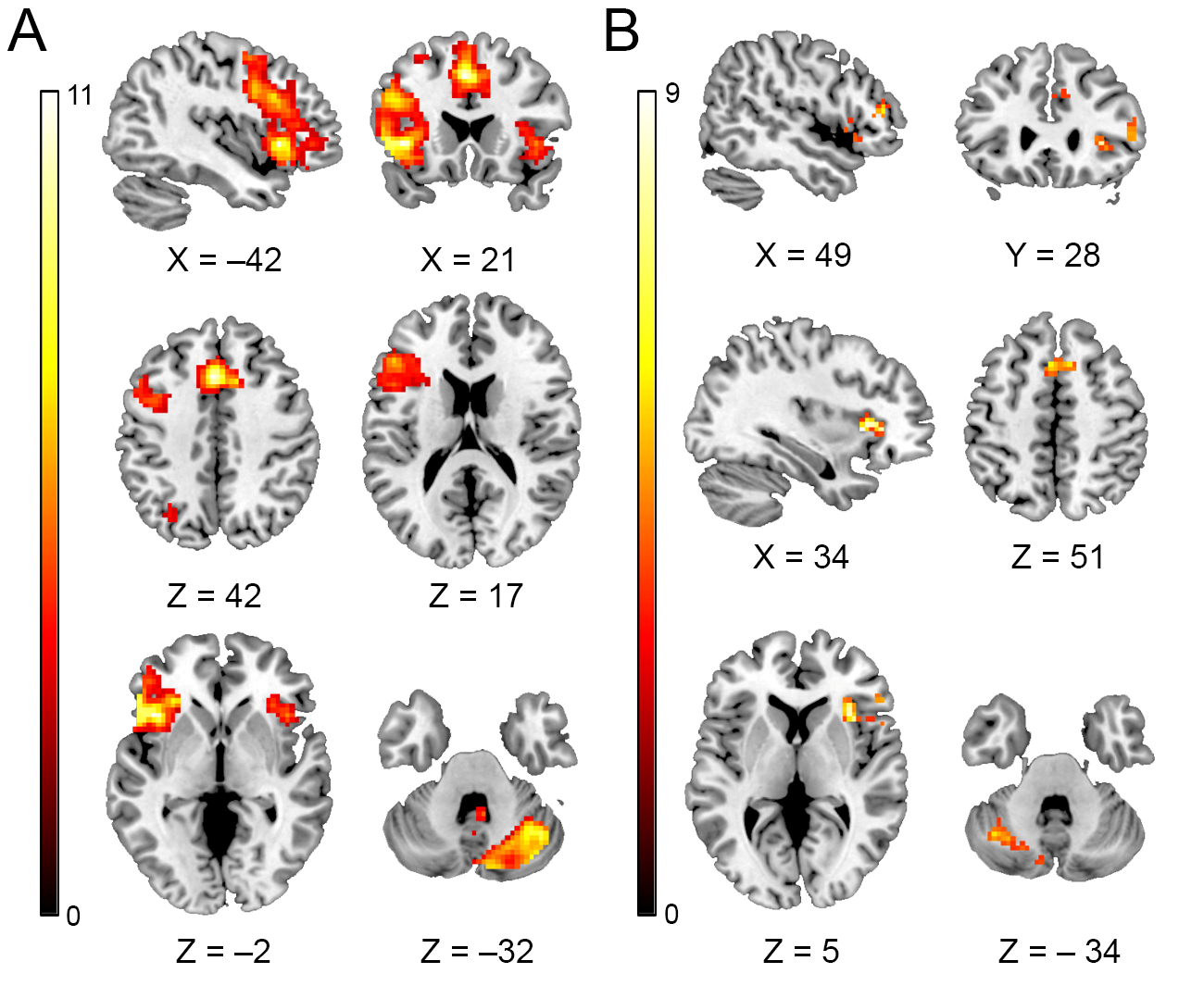

Supplement: Supplementary file 4 — Supplementary Figure 3 Brain activation maps resulting from the activation > control contrast during the verb generation task. Voxel‐wise threshold at p < 0.001, FWE cluster‐corrected at p < 0.05, coordinates reported in MNI space, color bars represent t values. (A) Left‐lateralized group. (B) Right‐lateralized group. Between‐groups parallelism of task‐related activations is not only evident in the inferior frontal area, but also in the cerebelum, which supports our lateralization assessment. It should be noted that, when using a less strict threshold in the right‐lateralized group (voxel‐wise threshold of p < 0.005; FWE corrected at p < 0.05), insula activity was also found in the left hemisphere, thus matching the pattern observed in the left‐lateralized group, and pointing to the pars triangularis cluster as the truly lateralized frontal activity. Activation clusters comprising the pars triangularis of the IFG (right for right‐lateralizeds, left for left‐lateralizeds) were used as seeds for the resting‐state functional connectivity analyses. [file HBM-41-2048-s004.tif]

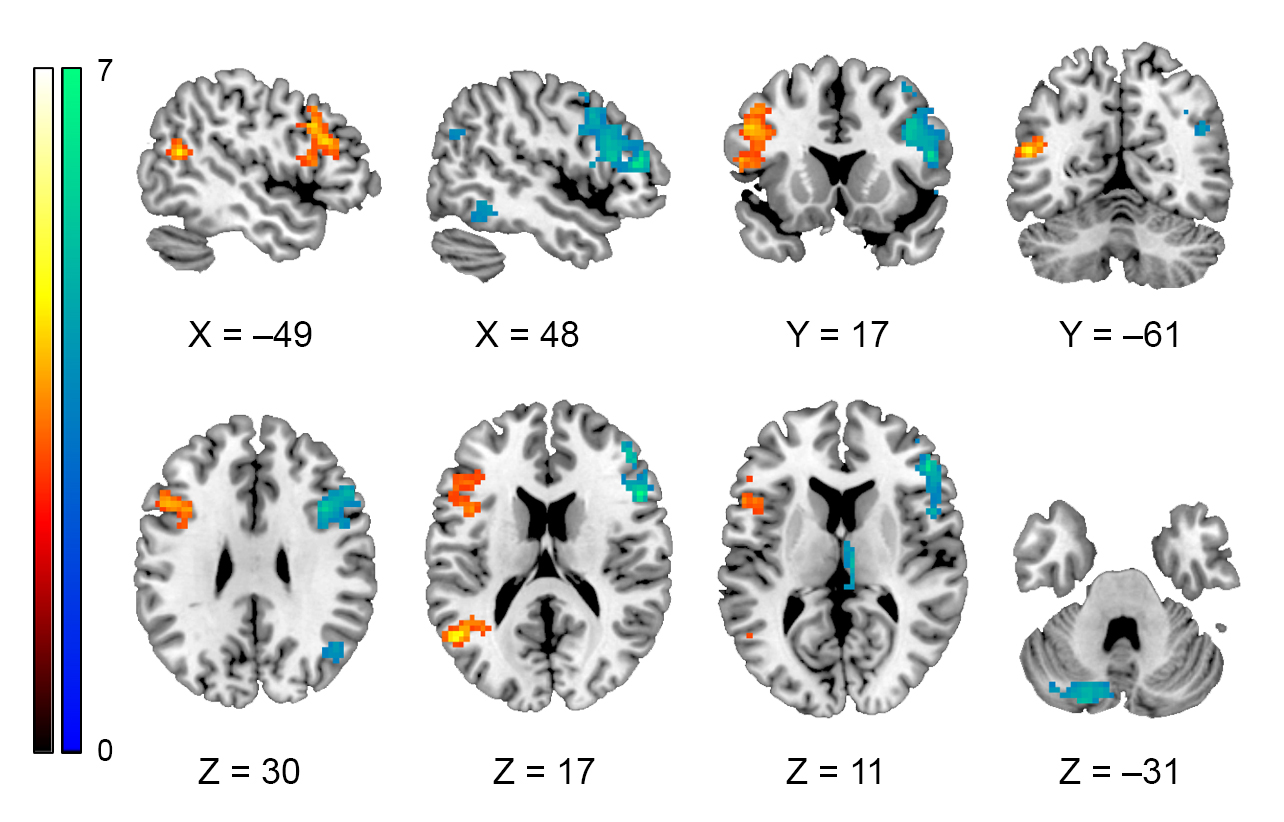

Supplement: Supplementary file 5 — Supplementary Figure 4 Differences in brain activity during the verb generation task according to language lateralization. Voxel‐wise threshold at p < 0.001, FWE cluster‐corrected at p < 0.05, coordinates reported in MNI space, color bars represent t values. Left‐lateralized participants > right‐lateralized participants (hot colors); and right‐lateralized participants > left‐lateralized participants (cold colors). Note that there are no differences in the anterior insula regions depicted in the one‐samples, thus confirming that anterior lateralization differences are confined to the pars triangularis. [file HBM-41-2048-s005.tif]
