# Supplementary material for: Left‐handed musicians show a higher probability of atypical cerebral dominance for language
Source: Hum Brain Mapp. 2020 Feb 7;41(8):2048–58. doi: 10.1002/hbm.24929 (PMC7268010; doi:10.1002/hbm.24929)
Supplement: Supplementary file 6 — Supplementary Table 1 Data used in the calculation of Laterality Indexes (LI). Note that unsmoothed images were used. ID = identification; M = musician; NM = non‐musician; k = voxel count (ROI); t = peak t value (ROI). [file HBM-41-2048-s006.docx]

| ID | group | Left hemisphere | | Right hemisphere | | LI |
| --- | --- | --- | --- | --- | --- | --- |
|  |  | *k* | *t* | *k* | *t* |  |
|  |  |  |  |  |  |  |
| 1 | NM | 306 | 15.29 | - | - | 100 |
| 2 | M | 371 | 12.02 | - | - | 100 |
| 3 | M | 339 | 11.96 | - | - | 100 |
| 4 | NM | 186 | 8.45 | - | - | 100 |
| 5 | NM | 159 | 8.02 | - | - | 100 |
| 6 | M | 285 | 7.62 | - | - | 100 |
| 7 | NM | 138 | 7.45 | - | - | 100 |
| 8 | M | 186 | 7.42 | - | - | 100 |
| 9 | NM | 38 | 7.16 | - | - | 100 |
| 10 | M | 88 | 7.11 | - | - | 100 |
| 11 | M | 25 | 6.89 | - | - | 100 |
| 12 | M | 135 | 6.82 | - | - | 100 |
| 13 | M | 247 | 6.63 | - | - | 100 |
| 14 | NM | 140 | 6.6 | - | - | 100 |
| 15 | NM | 311 | 6.21 | - | - | 100 |
| 16 | NM | 149 | 6.01 | - | - | 100 |
| 17 | M | 221 | 5.85 | - | - | 100 |
| 18 | NM | 18 | 5.72 | - | - | 100 |
| 19 | M | 117 | 5.54 | - | - | 100 |
| 20 | NM | 13 | 5.05 | - | - | 100 |
| 21 | NM | 39 | 4.62 | - | - | 100 |
| 22 | M | 70 | 3.8 | - | - | 100 |
| 23 | M | 68 | 3.66 | - | - | 100 |
| 24 | NM | 74 | 4.96 | - | - | 100 |
| 25 | M | 22 | 3.75 | - | - | 100 |
| 26 | M | 184 | 8.38 | 14 | 5.02 | 85.9 |
| 27 | NM | 423 | 9.76 | 33 | 6.06 | 85.5 |
| 28 | M | 394 | 7.21 | 35 | 5.29 | 83.7 |
| 29 | NM | 231 | 9.74 | 28 | 5.72 | 78.4 |
| 30 | NM | 285 | 7.94 | 36 | 5.98 | 77.6 |
| 31 | M | 160 | 6.14 | 23 | 3.9 | 74.9 |
| 32 | NM | 434 | 7.04 | 115 | 7.05 | 58.1 |
| 33 | M | 178 | 8.89 | 50 | 4.83 | 56.1 |
| 34 | NM | 170 | 6.54 | 48 | 5.23 | 56 |
| 35 | NM | 436 | 8.63 | 125 | 5.56 | 55.4 |
| 36 | NM | 290 | 6.43 | 85 | 5.58 | 54.7 |
| 37 | M | 417 | 8.66 | 370 | 8.9 | 6 |
| 38 | M | 135 | 5.34 | 376 | 5.86 | –47.2 |
| 39 | M | 74 | 4.54 | 229 | 5.52 | –51.2 |
| 40 | M | 98 | 6.8 | 379 | 9.87 | –58.9 |
| 41 | M | 67 | 4.46 | 304 | 4.69 | –63.9 |
| 42 | M | 21 | 4.66 | 132 | 5.61 | –72.5 |
| 43 | NM | 28 | 5.07 | 327 | 8.52 | –84.2 |
| 44 | M | - | - | 29 | 5.09 | –100 |
| 45 | M | - | - | 14 | 5.4 | –100 |
| 46 | M | - | - | 10 | 5.19 | –100 |
| 47 | M | - | - | 3 | 5.28 | –100 |
| 48 | M | - | - | 15 | 5.76 | –100 |
| 49 | M | - | - | 281 | 6.26 | –100 |
|  |  |  |  |  |  |  |

**Table S1**. Data used in the calculation of Laterality Indexes (LI). Note that unsmoothed images were used. ID = identification; M = musician; NM = non-musician; *k* = voxel count (ROI); *t* = peak *t* value (ROI).
