# Supplementary material for: Left‐handed musicians show a higher probability of atypical cerebral dominance for language
Source: Hum Brain Mapp. 2020 Feb 7;41(8):2048–58. doi: 10.1002/hbm.24929 (PMC7268010; doi:10.1002/hbm.24929)
Supplement: Supplementary file 7 — Supplementary Table 2 Cerebral activations of left‐lateralized and right‐lateralized groups during the verb generation task. Voxel‐wise threshold at p < 0.001, FWE cluster‐corrected at p < 0.05, coordinates reported in the MNI space. L = left, R = right. [file HBM-41-2048-s007.docx]

**Table S2**. Cerebral activations of left-lateralized and right-lateralized groups during the verb generation task. Voxel-wise threshold at *p* < 0.001, FWE cluster-corrected at *p* < 0.05, coordinates reported in the MNI space. L = left, R = right.

| Region  (peak) | BA  (cluster) | *k* | X | Y | Z | *t*- value  (peak) |
| --- | --- | --- | --- | --- | --- | --- |
| *a) Left-lateralized* | | | | | | |
| R cerebelum crus 2 | - | 500 | 18 | −82 | −37 | 11.48 |
| L anterior insula | 6, 8, 32, 44, 45, 47, 48 | 1984 | −42 | 20 | −1 | 11.29 |
| R anterior insula | 47, 48 | 140 | 42 | 20 | −1 | 6.54 |
| R cerebelum vermis | - | 40 | 6 | −49 | −31 | 5.99 |
| L angular gyrus | 7 | 33 | −30 | −67 | 41 | 4.42 |
| *b) Right-lateralized* | | | | | | |
| R anterior insula | 47, 48 | 68 | 33 | 17 | 2 | 9.41 |
| R SMA | 6, 32 | 121 | 9 | 11 | 59 | 7.82 |
| L cerebelum vermis | - | 36 | 0 | −52 | −19 | 7.68 |
| L cerebelum crus 1 | - | 91 | −36 | −55 | −37 | 6.95 |
| R pars triangularis | 45 | 27 | 48 | 38 | 11 | 6.86 |
