# Supplementary material for: Left‐handed musicians show a higher probability of atypical cerebral dominance for language
Source: Hum Brain Mapp. 2020 Feb 7;41(8):2048–58. doi: 10.1002/hbm.24929 (PMC7268010; doi:10.1002/hbm.24929)
Supplement: Supplementary file 8 — Supplementary Table 3 Differences between left‐lateralized and right‐lateralized groups in brain activity during the verb generation task. Voxel‐wise threshold at p < 0.001, FWE cluster‐corrected at p < 0.05, coordinates reported in the MNI space. L = left, R = right. [file HBM-41-2048-s008.docx]

**Table S3**. Differences between left-lateralized and right-lateralized groups in brain activity during the verb generation task. Voxel-wise threshold at *p* < 0.001, FWE cluster-corrected at *p* < 0.05, coordinates reported in the MNI space. L = left, R = right.

| Region  (peak) | BA  (cluster) | *k* | X | Y | Z | *t*- value  (peak) |
| --- | --- | --- | --- | --- | --- | --- |
| *a) Left-lateralized* > *Right-lateralized* | | | | | | |
| L middle frontal gyrus | 44, 45, 46, 48 | 245 | −42 | 20 | 41 | 7.2 |
| L angular gyrus | 21, 37, 39 | 63 | −39 | −52 | 20 | 5.64 |
| L precentral gyrus | 6, 8, 9 | 38 | −36 | −4 | 59 | 4.53 |
| *b) Right-lateralized* > *Left-lateralized* | | | | | | |
| R pars triangularis | 6, 8, 9, 44, 45, 48 | 457 | 51 | 20 | 17 | 6.6 |
| L cerebellum crus 1 | - | 77 | −15 | −76 | −28 | 5.14 |
| R thalamus | - | 31 | 6 | −19 | 11 | 4.74 |
| R inferior temporal gyrus | 20, 37 | 28 | 51 | −49 | −13 | 4.51 |
| R angular gyrus | 7, 19, 39, 40 | 58 | 36 | −73 | 35 | 4.1 |
