# Supplementary material for: Left‐handed musicians show a higher probability of atypical cerebral dominance for language
Source: Hum Brain Mapp. 2020 Feb 7;41(8):2048–58. doi: 10.1002/hbm.24929 (PMC7268010; doi:10.1002/hbm.24929)
Supplement: Supplementary file 9 — Supplementary Table 4 Differences between left‐lateralized and right‐lateralized groups in seed‐based resting‐state functional connectivity. Voxel‐wise threshold at p < 0.001, FWE cluster‐corrected at p < 0.05, coordinates reported in the MNI space. L = left, R = right. [file HBM-41-2048-s009.docx]

**Table S4**. Differences between left-lateralized and right-lateralized groups in seed-based resting-state functional connectivity. Voxel-wise threshold at *p* < 0.001, FWE cluster-corrected at *p* < 0.05, coordinates reported in the MNI space. L = left, R = right.

| Region  (peak) | BA  (cluster) | *k* | X | Y | Z | *t*- value  (peak) | | *z- value mean* ± SD  (cluster) |
| --- | --- | --- | --- | --- | --- | --- | --- | --- |
| *a) SEED: R pars triangularis / Left-lateralized* > *Right-lateralized* | | | | | | | |  |
| L middle frontal gyrus | 9, 45, 46 | 132 | −27 | 42 | 27 | | 7.1 | 0.04 ± 0.11 > −0.24 ± 0.09 |
| R middle frontal gyrus | 46 | 58 | 24 | 45 | 18 | | 4.98 | 0.12 ± 0.16 > −0.13 ± 0.12 |
| L anterior insula | 45, 48 | 51 | −42 | 12 | −3 | | 4.48 | 0.16 ± 0.16 > −0.1 ± 0.17 |
| *b) SEED: R pars triangularis / Right-lateralized* > *Left-lateralized* | | | | | | | |  |
| R cerebellum IX | - | 30 | 9 | −51 | −45 | | 5.55 | 0.04 ± 0.10 > −0.19 ± 0.10 |
| L lingual gyrus | 17, 30 | 89 | −6 | −57 | 6 | | 4.81 | 0.03 ± 0.15 > −0.24 ± 0.16 |
| R angular gyrus | 39 | 30 | 54 | −66 | 30 | | 3.82 | 0.11 ± 0.19 > −0.17 ± 0.18 |
